# Subclinical/overt hypothyroidism may be associated with diminished ovarian reserve in infertile women independent of thyroid autoimmunity

**DOI:** 10.3389/fendo.2024.1477665

**Published:** 2024-12-10

**Authors:** Hongzhan Zhang, Han Qiu, Zhiqiang Liu, Yulian Wu, Wei Liu, Chunyu Huang

**Affiliations:** ^1^ Shenzhen Key Laboratory of Reproductive Immunology for Peri-implantation, Shenzhen Zhongshan Institute for Reproductive Medicine and Genetics, Shenzhen, China; ^2^ Department of Reproductive Immunology, Guangdong Engineering Technology Research Center of Reproductive Immunology for Peri-implantation, Shenzhen, China; ^3^ Fertility Center, Shenzhen Zhongshan Obstetrics & Gynecology Hospital (formerly Shenzhen Zhongshan Urology Hospital), Shenzhen, China

**Keywords:** infertility, hypothyroidism, thyroid stimulating hormone, thyroid autoimmunity, ovarian reserve, anti-Müllerian hormone

## Abstract

**Objective:**

To investigate the association between thyroid dysfunction or thyroid autoimmunity (TAI) and diminished ovarian reserve (DOR).

**Methods:**

A total of 2,867 women undergoing their first *in-vitro* fertilization (IVF) cycle at Shenzhen Zhongshan Obstetrics & Gynecology Hospital between January 1, 2013 and June 30, 2021, were enrolled in this study. The participants had documented thyroid and ovarian reserve metrics. They were categorized into three groups based on their thyroid function: normal thyroid function (N = 2,540), subclinical/overt hypothyroidism (SCH/OH) (N = 290), and subclinical/overt hyperthyroidism (N = 37). Anti-Mullerian hormone (AMH) and antral follicle count (AFC) were assessed and collected. Women with AMH <1.2 ng/mL and AFC < 5 were diagnosed with DOR. Basic characteristics and ovarian reserve-related parameters were compared among the three groups. The association between thyroid function and ovarian reserve function was further analyzed using logistical regression analyses. In addition, the euthyroid population was stratified using a thyroid-stimulating hormone (TSH) threshold of 2.5 µIU/mL, and the ovarian reserve-related parameters were compared among women with low-normal TSH (TSH < 2.5 µIU/mL), high-normal TSH (2.5 µIU/mL ≤ TSH ≤ 4.2 µIU/mL) and SCH/OH.

**Results:**

Women with SCH/OH had lower AMH levels (2.79 ng/mL vs. 3.41 ng/mL, *P* < 0.001) and a significantly higher prevalence of AMH level < 1.2ng/mL (17.2% vs. 12.1%, *P* = 0.015) compared to those with normal thyroid function. The prevalence of DOR was also higher among women with SCH/OH (10.0% vs. 6.5%, *P* = 0.036). There were no significant differences in ovarian reserve between women with normal thyroid function and those with subclinical/overt hyperthyroidism. Logistic regression analyses showed that the odds ratio (OR) of women with SCH/OH suffering from DOR was 1.666 (95% CI: 1.079-2.572) compared to those with normal thyroid function, after adjusting for TAI status and basic clinical characteristics. When the euthyroid group was stratified according to TSH levels, women with SCH/OH showed significantly lower AMH levels compared to women with low-normal TSH (2.79 ng/mL vs. 3.44 ng/mL, *P* < 0.001) and a significantly higher prevalence of DOR (10.0% vs. 6.0%, *P* = 0.010). Logistic regression analyses showed that the women with SCH/OH had an increased prevalence of DOR (OR: 1.819, 95% CI: 1.158-2.858) compared to those with low-normal TSH, after adjusting for TAI status and basic clinical characteristics. However, the OR for DOR among women with high-normal TSH was not significantly elevated compared to those with low-normal TSH (OR: 1.310, 95% CI: 0.936-1.832).

**Conclusion:**

SCH/OH may be associated with DOR, irrespective of TAI status.

## 1 Introduction

Diminished ovarian reserve (DOR) is a reproductive condition characterized by a depletion of the ovarian follicle pool, resulting in a decreased number of eggs and/or a decline in the quality of the remaining eggs (1). DOR may reduce fertility in women attempting spontaneous conception or those undergoing assisted reproductive technologies (ART) such as *in vitro* fertilization (IVF) (2–4). Research into the etiology of this condition is crucial for improving diagnostic and therapeutic strategies for women suffering from DOR. Although the exact cause is multifactorial, studies have identified several contributing factors, such as genetic predisposition, environmental influences, and specific medical conditions (5–8). Recent studies have found that endocrine abnormalities, such as thyroid dysfunction, may be associated with DOR (9–11).

The thyroid is the largest endocrine gland in the human body and thyroid hormones, such as thyroid-stimulating hormone (TSH), free triiodothyronine (FT3) and free thyroid hormone (FT4), play a significant role in the growth and maturation of various organs (12–15). Thyroid dysfunction is a common endocrine disorder that significantly affects the fertility of women of reproductive age (16). Hyperthyroidism, characterized by elevated FT4 levels and decreased TSH levels, often manifests with menstrual irregularities such as amenorrhea, hypomenorrhea, and oligomenorrhea (17, 18). Overt hypothyroidism (OH) and subclinical hypothyroidism (SCH), identified by elevated levels of TSH, have been associated with miscarriage and preterm birth (19). Interestingly, thyroid hormone and TSH receptors have been observed on granulosa cells and oocytes in the ovary, where they act either directly or synergistically with follicle-stimulating hormone (FSH) to influence follicular growth and development (20). This finding has drawn attention to the relationship between thyroid dysfunction and DOR. However, the conclusions on this relationship remain controversial (21–24). For instance, Michalakis et al. found that elevated preconception levels of TSH were associated with DOR (21). However, Kucukler et al. found that ovarian reserve function was not different among women with OH, SCH, and normal thyroid function (23).

Previous studies also demonstrated that women who were diagnosed with thyroid autoimmunity (TAI) are more likely to develop thyroid dysfunction, such as OH or SCH (25, 26). Some studies have indicated that TAI is also a risk factor for impaired ovarian reserve function (27, 28). The complex link between TAI and thyroid dysfunction makes it difficult to distinguish their independent effect on ovarian reserve function, and most previous studies have ignored the interaction between these two factors (23, 29), limiting the clarity of their respective roles in ovarian reserve function.

In light of these considerations, this study aimed to explore the relationship between thyroid dysfunction and DOR in infertile women. Additionally, the potential modifying effect of TAI on this relationship was evaluated.

## 2 Materials and methods

### 2.1 Ethical approval

The study was approved by the Ethics Committee of Shenzhen Zhongshan Obstetrics & Gynecology Hospital (formerly Shenzhen Zhongshan Urology Hospital) (approval number: 2023SZM-LW006). A waiver of informed consent was granted for this study.

### 2.2 Subjects

A cross-sectional study was conducted on infertile women who underwent ART at Shenzhen Zhongshan Obstetrics & Gynecology Hospital (formerly Shenzhen Zhongshan Urology Hospital) between January 1, 2013 and June 30, 2021.

The inclusion criteria were as follows: (1) women aged 20-40 years old; (2) women undergoing their first IVF/ICSI treatment at our hospital; (3) women with integrated electronic medical record data; and (4) women who met the criteria for normal thyroid function, OH, SCH, overt hyperthyroidism or subclinical hyperthyroidism.

The exclusion criteria for women in this study were as follows: a history of medication use, including thyroid hormone, anti-thyroid medication, and thyroid surgery (N = 24); a diagnosis of polycystic ovarian syndrome, endometriosis, or ovarian tumor (N = 689); a history of chemotherapy, radiotherapy, or ovarian surgery (N = 222); and X chromosome abnormalities (N = 18).

**Figure 1:**
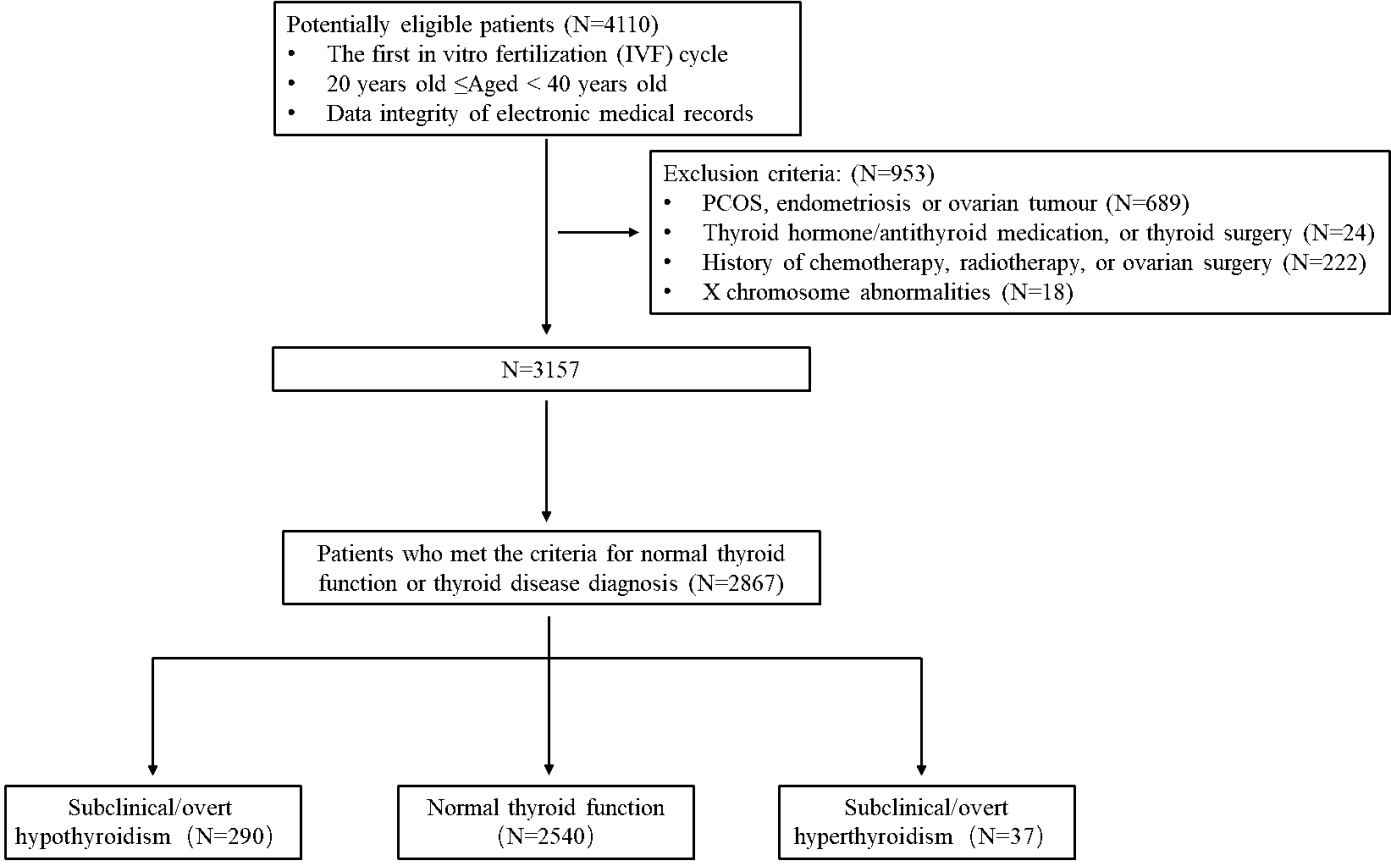
Diagram of the study and distribution of the patients investigated.

To ensure the accuracy of the study, we thoroughly examined each electronic record to confirm that all participants met the specified inclusion and exclusion criteria. Finally, 2867 women were enrolled in the analysis (**Figure 1**). Based on their TSH and FT4 levels, the participants were categorized into three groups: those with normal thyroid function (N = 2,540), SCH/OH (N = 290), and those with subclinical/overt hyperthyroidism (N = 37).

### 2.3 Variable measurement and collection procedures

#### 2.3.1 Demographic characteristics

Height and weight were measured for all patients. In addition, detailed medical background information was collected, including age, duration of infertility, and type of infertility.

#### 2.3.2 Sex hormones

Endocrine hormones, including FSH, luteinizing hormone (LH), and estradiol (E2), were measured using a chemiluminescence immunoassay (Roche Diagnostics, Germany) conducted on the 2nd to 5th day of the menstrual cycle. These measurements were taken before ovarian stimulation, following standard protocols. In our laboratory, the reference ranges for basal hormone levels were as follows: FSH, 3.5 to 12.5 IU/L, LH, 2.4 to 12.6 IU/L, and E2, 0 to 160 pg/mL, as provided by the manufacturer. The sensitivity threshold for measuring FSH and LH was set at 0.1 IU/L.

#### 2.3.3 Thyroid function examination

Thyroid function parameters, including TSH, FT4, and FT3, were measured using the electrochemical luminescence method on a Cobas E601 analyzer (Roche Diagnostics GmbH, Germany). Thyroid peroxidase antibody (TPOAb) and thyroglobulin antibody (TgAb) were assessed using a commercial chemiluminescence immunoassay (YHLO Biotech, China). The minimum detectable levels for TPOAb and TgAb were 0.1 IU/mL, with an intra-assay coefficient of variation below 10%. The reference ranges for thyroid function tests were as follows, TSH: 0.27-4.20 μIU/mL, FT3: 2.0-4.4 pg/mL, and FT4: 0.93-1.70 ng/mL. The sensitivity threshold for measuring TSH was 0.005 μIU/mL. A TPOAb titer above 34 IU/mL or a TgAb titer above 115 IU/mL was considered positive, and positivity for either or both antibodies was used to define TAI. Thyroid dysfunction was categorized as follows: OH, TSH > 4.2 mIU/L with FT4 < 0.93 ng/mL; SCH, TSH > 4.2 mIU/L with FT4 levels within the normal range; overt hyperthyroidism, TSH < 0.1 mIU/L with FT4 > 1.7 ng/mL or FT3 > 4.4 pg/mL; and subclinical hyperthyroidism, TSH < 0.1 mIU/L with FT4 and FT3 levels within the normal range.

#### 2.3.4 Ovarian reserve function parameters

Serum anti-Mullerian hormone (AMH) levels were measured using a commercial chemiluminescence immunoassay (YHLO Biotech, China). In statistical analysis, AMH values below the detection limit (0.01ng/mL) were recorded as 0.01 ng/mL. Antral follicle count (AFC) referred to the total number of early sinusoidal follicles (2-10 mm independent diameter) observed via transvaginal ultrasound on both ovaries.

### 2.4 Outcomes

This study analyzed the prevalence of women with DOR, defined as an AMH level < 1.2 ng/mL and AFC level < 5. In addition, the prevalence of women with an AMH level < 1.2 ng/mL regardless of AFC level, and those with an AFC level < 5 regardless of AMH level, was examined.

### 2.5 Statistical analyses

All statistical analyses and graphical representations were performed using SPSS version 26.0 and R version 4.2.1. The normality of continuous variables was assessed by combining the Kolmogorov-Smirnov test and quantile-quantile plot. For continuous variables with non-normal distribution, data were presented as medians (with interquartile ranges) and compared using non-parametric tests. The Kruskal-Wallis rank-sum test was used to compare continuous variables across three groups, with pairwise comparisons performed using Dunn’s with Bonferroni correction. Qualitative variables were presented as absolute values and percentages (%). Differences in outcomes between qualitative variables were analyzed using the chi-square test, while pairwise comparisons among multiple groups were conducted using the chi-square partitioning method. Fisher’s exact probability test was used when the expected frequency was less than 5.

To assess the impact of thyroid dysfunction and TAI on ovarian reserve, binary and multinomial logistic regression models were employed to calculate odds ratios (OR) and 95% confidence intervals (CI). The logistic regression models were adjusted for potential confounders, including TAI, female age, BMI, infertility duration and infertility type. A *P-*value of 0.05 was considered statistically significant.

## 3 Results

### 3.1 Prevalence of DOR was increased in infertile women with SCH/OH compared to women with normal thyroid function

To investigate the differences in ovarian reserve function among women with different thyroid conditions, we compared ovarian reserve parameters among infertile women with normal thyroid function, SCH/OH, and subclinical/overt hyperthyroidism (**Table 1**). The concentration of TPOAb (9.33 IU/mL vs. 32.90 IU/mL vs. 6.35 IU/mL, *P* < 0.001) and TgAb (14.69 IU/mL vs. 108.3 IU/mL vs. 12.55 IU/mL, *P* < 0.001) was significantly increased in the women with SCH/OH and subclinical/overt hyperthyroidism compared to those with normal thyroid function. Similarly, the prevalence of TPOAb (27.2% vs. 48.6% vs. 10.3%, *P* < 0.001) and TgAb (28.3% vs. 48.6% vs. 12.9%, *P* < 0.001) was notably elevated among women with SCH/OH and subclinical/overt hyperthyroidism compared to women with normal thyroid function.

**Table 1:** Baseline characteristics and ovarian reserve parameters of participants with different thyroid conditions.

Variables	Normal thyroid function	SCH/OH	Subclinical/overt hyperthyroidism	*P*
N	2540	290	37	
Age (years)	32.00 [29.00, 35.00]	32.00 [29.00, 35.00]	31.00 [29.00, 34.00]	0.139
BMI (kg/m^2^)	20.81 [19.31, 22.66]	21.36 [20.03, 24.03]	21.08 [19.47, 23.24]	<0.001^a^
Duration of infertility (years)	3.00 [2.00, 5.00]	3.00 [2.00, 5.00]	3.00 [2.00, 5.00]	0.657
Infertility type, n (%)				
Primary infertility	1178 (46.4)	156 (53.8)	24 (64.9)	0.006^a^
Secondary infertility	1362 (53.6)	134 (46.2)	13 (35.1)	
TSH (µIU/mL)	1.95 [1.42, 2.68]	5.08 [4.54, 6.34]	0.02 [0.00, 0.03]	<0.001^a,b,c^
FT4 (ng/dL)	1.25 [1.14, 1.35]	1.20 [1.10, 1.30]	1.77 [1.44, 2.11]	<0.001^a,b,c^
FT3 (pg/mL)	2.94 [2.73, 3.17]	2.99 [2.75, 3.20]	4.14 [3.21, 5.06]	<0.001^b, c^
TPOAb (IU/mL)	6.35 [1.68, 12.27]	9.33 [3.42, 42.90]	32.90 [14.13, 143.10]	<0.001^a,b,c^
TgAb (IU/mL)	12.55 [5.23, 23.27]	14.69 [7.93, 187.00]	108.30 [15.30, 696.80]	<0.001^a,b,c^
Positive TPOAb, n(%)	261 (10.3)	79 (27.2)	18 (48.6)	<0.001^a, b,c^
Positive TgAb, n(%)	327 (12.9)	82 (28.3)	18 (48.6)	<0.001^a,b,c^
Positive TAI, n(%)	396 (15.6)	95 (32.8)	19 (51.4)	<0.001^a,b,c^
LH (IU/L)	4.91 [3.76, 6.41]	4.80 [3.59, 6.45]	4.86 [3.90, 6.07]	0.948
E2 (pg/mL)	34.90 [26.00, 47.50]	32.60 [24.15, 45.65]	41.10 [33.60, 58.80]	0.004^b,c^
AMH (ng/mL)	3.41 [1.94, 5.47]	2.79 [1.46, 4.82]	3.64 [1.54, 4.91]	0.002^a^
AFC (n)	10.00 [6.00, 14.00]	9.00 [6.25, 14.00]	9.00 [6.00, 14.00]	0.849
FSH (mIU/mL)	6.78 [5.85, 8.01]	7.03 [6.06, 8.09]	6.72 [5.75, 7.69]	0.237
Prevalence of AMH< 1.2 ng/mL, n(%)	308 (12.1)	50 (17.2)	5 (13.5)	0.045^a^
Prevalence of AFC<5, n(%)	329 (13.0)	43 (14.8)	3 (8.1)	0.445
Prevalence of DOR, n(%)	166 (6.5)	29 (10.0)	1 (2.7)	0.052

All data is presented as median [25th percentile, 75th percentile] or n (%).

Superscripts indicate significant differences between ^a^Normal thyroid function and SCH/OH, ^b^ SCH/OH and subclinical/overt hyperthyroidism, and ^c^Normal thyroid function and subclinical/overt hyperthyroidism.

Kruskal-Wallis rank-sum test is applied for comparisons among three groups of continuous variables, with pairwise comparisons via Dunn’s with Bonferroni correction. Categorical variables are compared using chi-squared test, with pairwise comparisons among multiple groups are conducted using the chi-square partitioning method.

SCH/OH, Subclinical/overt hypothyroidism; BMI, body–mass index; TSH, thyroid stimulating hormone; FT4, free thyroxine; FT3, free triiodothyronine; TPOAb, thyroid peroxidase antibody; TgAb, thyroglobulin antibody; TAI, thyroid autoimmunity; LH, luteinizing hormone; E2, estradiol; AMH, anti-müllerian hormone; AFC, antral follicle count; FSH, follicle stimulating hormone; DOR, diminished ovarian reserve.

An elevated E2 level was observed predominantly in the subclinical/overt hyperthyroidism group (41.10 pg/mL vs. 34.90 pg/mL vs. 32.60 pg/mL, *P* = 0.004) compared to women with normal thyroid function and those with SCH/OH. Notably, AMH levels were significantly lower in women with SCH/OH compared to those with normal thyroid function (2.79 ng/mL vs. 3.41 ng/mL, *P* < 0.001). In addition, the prevalence of women with AMH <1.2 ng/mL was significantly higher in the SCH/OH group compared to the normal thyroid function group (17.2% vs. 12.1%, *P* = 0.015). Moreover, there was a noticeable trend toward a higher prevalence of DOR in women with SCH/OH compared to those with normal thyroid function (10.0% vs. 6.5%, *P* = 0.036).

### 3.2 SCH/OH may be associated with DOR in infertile women

Univariate logistic regression analysis showed a significant association between SCH/OH and DOR (OR: 1.589, 95% CI: 1.05-2.406; **Table 2**), while overt/subclinical hyperthyroidism was not related to the occurrence of DOR (OR: 0.397, 95% CI: 0.054-2.916). Interestingly, TAI positivity did not significantly increase the prevalence of DOR (OR: 1.005, 95% CI: 0.688-1.468).

**Table 2:** Effect of thyroid conditions for DOR.

Variables	Model 1 OR(95% CI)	Model 2 OR(95% CI)	Model 3 OR(95% CI)	Model 4 OR(95% CI)
Thyroid conditions				
Normal thyroid function	Ref		Ref	Ref
SCH/OH	1.589(1.050,2.406)^*^		1.598(1.050,2.432)^*^	1.666(1.079,2.572)^*^
Subclinical/overt hyperthyroidism	0.397(0.054,2.916)		0.402(0.055,2.965)	0.463(0.062,3.459)
Thyroid autoimmunity				
TAI(-)		Ref	Ref	Ref
TAI(+)		1.005(0.688,1.468)	0.966(0.658,1.420)	0.9(0.608,1.333)
Basic clinical characteristics				
Female age (years)				1.223(1.168,1.281)^*^
BMI (kg/m^2^)				1.025(0.973,1.079)
Duration of infertility (years)				1.015(0.968,1.065)
Infertility type				
Primary infertility				Ref
Secondary infertility				0.64(0.468,0.875)^*^

Model 1: Univariate logistic regression analysis of thyroid function. Model 2: Univariate logistic regression analysis of TAI. Model 3: Adjusted for thyroid autoimmunity. Model 4: Model 3, with additional adjustments for female age, BMI, infertility duration, infertility type.

*P value<0.05.

DOR, diminished ovarian reserve; SCH/OH, Subclinical/overt hypothyroidism; TAI, thyroid autoimmunity.

Multivariate logistic regression analyses indicated that SCH/OH remained significantly associated with DOR after adjusting for confounding factors, including TAI status, female age, BMI, duration of infertility, and type of infertility (OR: 1.666, 95% CI: 1.079-2.572).

To further analyze the impact of TAI status on the relationship between SCH/OH and DOR, interaction analyses were performed (**Table 3**). Interaction analyses showed that SCH/OH alone was significantly associated with DOR (adjusted OR: 1.839; 95% CI: 1.116-3.031). In contrast, the presence of TAI alone was not significantly associated with DOR (adjusted OR: 0.996; 95% CI: 0.645-1.538). In addition, no significant interaction was found between TAI and thyroid conditions (adjusted OR: 1.174; 95% CI: 0.550-2.508).

**Table 3:** Modification of association between different thyroid conditions and DOR by thyroid autoimmunity.

Variables	Number of patients	Prevalence of DOR, n(%)	Adjusted OR (95% CI)
TAI(-)			
Normal thyroid function	2144	139(6.48)	Ref
SCH/OH	195	21(10.77)	1.871(1.133,3.089)^*^
TAI(+)			
Normal thyroid function	396	27(6.82)	Ref
SCH/OH	95	8(8.42)	1.126(0.472,2.687)
TAI&Thyroid condition			
TAI(-)&Normal thyroid function	2144	139(6.48)	Ref
TAI(-)&SCH/OH	195	21(10.77)	1.839(1.116,3.031) ^*^
TAI(+)&Normal thyroid function	396	27(6.82)	0.996(0.645,1.538)
TAI(+)&SCH/OH	95	8(8.42)	1.247(0.581,2.676)
Interaction of TAI and Thyroid condition			1.174(0.550,2.508)

Adjusted OR: Adjusted for female age, BMI, infertility duration, infertility type.

*P value<0.05.

DOR, diminished ovarian reserve; SCH/OH, Subclinical/overt hypothyroidism; TAI, thyroid autoimmunity.

### 3.3 Prevalence of DOR was similar between women with high-normal TSH and low-normal TSH

Owing to the limited number of cases with overt or subclinical hyperthyroidism, we opted not to conduct further stratified analyses for these conditions. Instead, women with normal thyroid function were divided into two groups, low-normal TSH and high-normal TSH, based on a threshold of 2.5 µIU/mL. It was found that TPOAb (9.33 IU/mL vs. 7.31 IU/mL vs. 5.95 IU/mL, *P* < 0.001) and TgAb (14.69 IU/mL vs. 13.41 IU/mL vs. 12.16 IU/mL, *P* < 0.001) levels were markedly elevated in both the SCH/OH group and the high-normal TSH group compared to the low-normal TSH group (**Table 4**). In alignment with this, the prevalence of positive TPOAb (27.2% vs. 12.6% vs. 9.3%, *P* < 0.001) TgAb (28.3% vs. 17.0% vs. 11.1%, *P* < 0.001) and TAI (32.8% vs. 19.5% vs. 13.9%, *P* < 0.001) was markedly elevated in the SCH/OH and high-normal TSH groups compared to the low-normal TSH group.

**Table 4:** Clinical characteristics of participants with normal thyroid function and SCH/OH.

Variables	TSH<2.5µIU/mL	2.5µIU/mL≤TSH≤4.2µIU/mL	SCH/OH	*P*
N	1771	769	290	
Age (years)	32.00 [29.00, 35.00]	32.00 [29.00, 35.00]	32.00 [29.00, 35.00]	0.284
BMI (kg/m^2^)	20.76 [19.23, 22.58]	20.96 [19.47, 22.86]	21.36 [20.03, 24.03]	<0.001^b,c^
Duration of infertility (years)	3.00 [2.00, 5.00]	3.00 [2.00, 5.00]	3.00 [2.00, 5.00]	0.388
Infertility type, n (%)				
Primary infertility	815 (46.0)	363 (47.2)	156 (53.8)	0.049^c^
Secondary infertility	956 (54.0)	406 (52.8)	134 (46.2)	
TSH (µIU/mL)	1.62 [1.23, 2.02]	3.07 [2.76, 3.51]	5.08 [4.54, 6.34]	<0.001^a,b,c^
FT4 (ng/dL)	1.25 [1.15, 1.36]	1.24 [1.13, 1.34]	1.20 [1.10, 1.30]	<0.001^b,c^
FT3 (pg/mL)	2.93 [2.71, 3.14]	2.98 [2.76, 3.23]	2.99 [2.75, 3.20]	<0.001^a^
TPOAb (IU/mL)	5.95 [1.51, 11.72]	7.31 [2.41, 13.89]	9.33 [3.42, 42.90]	<0.001^a,b,c^
TgAb (IU/mL)	12.16 [4.50, 22.18]	13.41 [6.80, 34.15]	14.69 [7.93, 187.00]	<0.001^c^
Positive TPOAb, n(%)	164 (9.3)	97 (12.6)	79 (27.2)	<0.001^a,b,c^
Positive TgAb, n(%)	196 (11.1)	131 (17.0)	82 (28.3)	<0.001^a,b,c^
Positive TAI, n(%)	246 (13.9)	150 (19.5)	95 (32.8)	<0.001^a,b,c^
LH (IU/L)	4.85 [3.69, 6.34]	5.02 [3.90, 6.57]	4.80 [3.59, 6.45]	0.069
E2 (pg/mL)	35.00 [26.00, 47.30]	34.00 [26.20, 48.00]	32.60 [24.15, 45.65]	0.087
AMH (ng/mL)	3.44 [1.96, 5.58]	3.33 [1.91, 5.23]	2.79 [1.46, 4.82]	<0.001^c^
AFC (n)	10.00 [6.00, 14.00]	10.00 [6.00, 14.00]	9.00 [6.25, 14.00]	0.852
FSH (mIU/mL)	6.72 [5.82, 7.98]	6.93 [5.90, 8.14]	7.03 [6.06, 8.09]	0.062
Prevalence of AMH< 1.2 ng/mL, n(%)	204 (11.5)	104 (13.5)	50 (17.2)	0.017^c^
Prevalence of AFC<5, n(%)	214 (12.1)	115 (15.0)	43 (14.8)	0.097
Prevalence of DOR, n(%)	106 (6.0)	60 (7.8)	29 (10.0)	0.022^c^

All data is presented as median [25th percentile, 75th percentile] or n (%).

Superscripts indicate significant differences between ^a^TSH<2.5µIU/mL and 2.5µIU/mL≤TSH≤4.2µIU/mL, ^b^2.5µIU/mL≤TSH≤4.2µIU/mL and SCH/OH, and ^c^TSH<2.5µIU/mL and SCH/OH.

Kruskal-Wallis rank-sum test is applied for comparisons among three groups of continuous variables, with pairwise comparisons via Dunn’s with Bonferroni correction. Categorical variables are compared using chi-squared test, with pairwise comparisons among multiple groups are conducted using the chi-square partitioning method.

SCH/OH, Subclinical/overt hypothyroidism; BMI, body–mass index; TSH, thyroid stimulating hormone; FT4, free thyroxine; FT3, free triiodothyronine; TPOAb, thyroid peroxidase antibody; TgAb, thyroglobulin antibody; TAI, thyroid autoimmunity; LH, luteinizing hormone; E2, estradiol; AMH, anti-müllerian hormone; AFC, antral follicle count; FSH, follicle stimulating hormone; DOR, diminished ovarian reserve.

AMH levels were significantly lower in the SCH/OH group compared with low-normal TSH group (2.79 ng/mL vs. 3.44 ng/mL, *P* < 0.001). Furthermore, the prevalence of women with AMH < 1.2 ng/mL (17.2% vs. 11.5%, *P* = 0.006) and those with DOR (10.0% vs. 6.0%, *P* = 0.01) was significantly higher in the SCH/OH group compared to the low-normal TSH group. However, no significant differences were observed in ovarian reserve-related parameters between the high-normal TSH group and the low-normal TSH group.

### 3.4 No association was found between high-normal TSH level and DOR

Univariate logistic regression indicated that SCH/OH was significantly associated with DOR (OR: 1.745, 95% CI: 1.134-2.686), while high-normal TSH level (OR: 1.329, 95% CI: 0.957-1.846) and TAI (OR: 1.045, 95% CI: 0.715-1.528) were not associated with DOR (**Table 5**). Multivariate logistic regression analysis after adjusting for confounding factors revealed that SCH/OH still remained significantly associated with an increased prevalence of DOR (OR: 1.819, 95% CI: 1.158-2.858). However, the OR for DOR in women with high-normal TSH level, compared to those with low-normal TSH levels, was not significantly elevated (OR: 1.310, 95% CI: 0.936-1.832). Furthermore, to eliminate the impact of OH, we also analyzed the data of women with low-normal TSH, high-normal TSH, and SCH. The conclusions were consistent with the aforementioned results (**Supplementary Tables S1**, **S2**).

**Table 5:** Effect of thyroid stimulating hormone levels on DOR.

Variables	Model 1 OR(95% CI)	Model 2 OR(95% CI)	Model 3 OR(95% CI)	Model 4 OR(95% CI)
TSH levels (µIU/mL)				
TSH<2.5	Ref		Ref	Ref
2.5≤TSH≤4.2	1.329(0.957,1.846)		1.332(0.958,1.851)	1.310(0.936,1.832)
SCH/OH	1.745(1.134,2.686)^*^		1.757(1.135,2.72)^*^	1.819(1.158,2.858)^*^
Thyroid autoimmunity				
TAI(-)		Ref	Ref	Ref
TAI(+)		1.045(0.715,1.528)	0.965(0.656,1.419)	0.902(0.609,1.336)
Basic clinical characteristics				
Female age (years)				1.219(1.164,1.277)^*^
BMI (kg/m^2^)				1.025(0.974,1.08)
Duration of infertility (years)				1.016(0.969,1.066)
Infertility type				
Primary infertility				Ref
Secondary infertility				0.649(0.475,0.888)^*^

Model 1: Univariate logistic regression analysis of TSH levels. Model 2: Univariate logistic regression analysis of TAI. Model 3: Adjusted for thyroid autoimmunity. Model 4: Model 3, with additional adjustments for female age, BMI, infertility duration, infertility type.

*P value<0.05.

DOR, diminished ovarian reserve; SCH/OH, Subclinical/overt hypothyroidism; TAI, thyroid autoimmunity.

## 4 Discussion

Thyroid dysfunction is a common endocrine disorder in women of reproductive age (30). This study aimed to investigate the effect of different thyroid conditions on ovarian reserve in infertile women while considering the status of TAI. The findings revealed that women with SCH/OH had significantly lower AMH levels and an increased prevalence of AMH < 1.2 ng/mL compared to women with normal thyroid function. Logistic regression analysis indicated that SCH/OH might be associated with DOR, while TAI did not affect this result. Furthermore, stratification of TSH levels in women with normal thyroid function revealed that the prevalence of DOR in women with SCH/OH was significantly higher than that in women with TSH < 2.5 µIU/mL, with no significant difference observed in women with TSH levels between 2.5 µIU/mL and 4.2 µIU/mL. These findings suggest that SCH/OH may have an independent effect on ovarian reserve.

SCH/OH, characterized by TSH levels exceeding 4.2 µIU/mL, negatively impact women’s reproductive health (23, 31). Previous study have shown that women with SCH had significantly lower AMH concentrations and AFCs compared to euthyroid women (32). Consistent with these findings, our study found that SCH/OH was associated with reduced AMH levels and an increased prevalence of AMH <1.2 ng/mL. This reduction in AMH levels was significantly associated with DOR, underscoring the direct impact of hypothyroid states on the reproductive potential of women. Extensive researches have established a robust connection between thyroid dysfunction and DOR (21, 33, 34). Hiraoka et al. reported a significantly higher prevalence of DOR in patients with elevated TSH levels (TSH ≥4.5 µIU/mL) compared to those with normal TSH levels (46.2% vs 17.9%, *P* < 0.05) (35). Similarly, our study found reduced AMH levels and a higher prevalence of DOR in patients with SCH/OH compared to euthyroid women. DOR may be associated with SCH/OH (TSH > 4.2 µIU/mL). Conversely, there was no association between hyperthyroidism or subclinical hyperthyroidism and DOR. This suggests that thyroid function may influence ovarian reserve, with the impact may be more pronounced in hypothyroidism rather than in hyperthyroidism.

TAI is commonly observed in women of reproductive age and stands as the primary etiology of thyroid dysfunction (36). Thyroid autoantibodies may directly damage ovarian tissue, leading to impaired follicular development and premature ovarian insufficiency (POI) (37). Research suggested that the presence of thyroid autoantibodies in women has been linked to the deposition of autoantibodies in their ovarian tissue (38). However, previous studies have reported conflicting findings concerning the relationship between TAI and ovarian reserve in euthyroid women (39–42). A large cohort study involving 21,325 participants demonstrated that TAI significantly increased the risk of POI in women, aligning with the outcomes of a recent meta-analysis (41, 43). Conversely, Rao et al. suggested that while TPOAb positivity was not associated with ovarian reserve, SCH was associated with a lower AMH concentration (mean difference = -0.27 ng/mL [CI -0.43 to -0.12 ng/mL], *P* = 0.001), and lower AFC (mean difference = -0.7 [CI -1.3 to -0.2], *P* = 0.005) (32). In our study, no significant association was observed between TAI and DOR. These findings suggested that while autoimmunity processes may influence thyroid function, their direct impact on ovarian reserve may be less pronounced or may involve different mechanisms that require further investigation.

Several studies have demonstrated the correlation between TSH and ovarian reserve in infertile populations (22, 44, 45). Weghofer et al. conducted a retrospective study on 225 infertile women with TSH levels within the normal range (0.4-4.5 mIU/L) and found that TSH <3.0 μIU/mL was significantly correlated with higher AMH levels (34). Halici et al. classified the TSH range into four categories: ≤1 mIU/L, 1-2.5 mIU/L, 2.5-4 mIU/L, and >4 mIU/L, and discovered that the association between TSH and AMH was not linear, with the highest AMH levels observed at a TSH value of 2.88 mIU/L. Conversely, significantly lower AMH levels were reported in the group with TSH >4 mIU/L compared to the other three groups (46). Consistent with these findings, our study found that women with SCH/OH (TSH >4.2 µIU/mL) had significantly lower AMH levels and a significantly higher prevalence of DOR compared to those with TSH < 2.5 µIU/mL. Even after adjusting for confounding variables such as TAI, SCH/OH remained a significant association with DOR. These results suggest that abnormally elevated TSH level may impair ovarian reserve, while lower TSH levels exert a direct beneficial effect on follicular recruitment.

This study had some limitations that should be acknowledged. The retrospective cross-sectional nature of the study limited the ability to establish a causal relationship between thyroid function and ovarian reserve. While the study provided valuable insights into the potential association further research utilizing different study designs, such as prospective longitudinal studies, is necessary to definitively determine the nature of this relationship. Additionally, all patients were recruited from a single center in the study, which raises the possibility of selection bias affecting the characteristics and outcomes of the participants. Future should consider recruiting participants from multiple centers to enhance the generalizability of the findings and minimize the potential impact of selection bias on the results.

## 5 Conclusions

Our findings revealed that SCH/OH may be associated with a reduction in ovarian reserve among infertile women and may act as an independent factor affecting ovarian reserve. While the presence of TAI may potentially dilute or obscure the impact of SCH/OH on ovarian reserve, SCH/OH still may be associated with DOR. In summary, thyroid dysfunction may adversely affect a woman’s fertility, highlighting the importance of early recognition and management of thyroid-related issues to address possible barriers to conception.

## Data Availability

The original contributions presented in the study are included in the article/**Supplementary Material**. Further inquiries can be directed to the corresponding author.
