# Supplementary material for: Subclinical/overt hypothyroidism may be associated with diminished ovarian reserve in infertile women independent of thyroid autoimmunity
Source: Front Endocrinol (Lausanne). 2024 Dec 10;15:1477665. doi: 10.3389/fendo.2024.1477665 (PMC11666349; doi:10.3389/fendo.2024.1477665)
Supplement: Supplementary file 1 [file Table1.docx]

**Supplemental Table 1** Baseline characteristics and ovarian reserve parameters of participants with normal thyroid function and SCH.

| **Variables** | **TSH<2.5µIU/mL** | **2.5µIU/mL≤TSH≤4.2µIU/mL** | **SCH** | ***P*** |
| --- | --- | --- | --- | --- |
| N | 1771 | 769 | 272 |  |
| Age (years) | 32.00 [29.00, 35.00] | 32.00 [29.00, 35.00] | 32.00 [29.00, 35.00] | 0.26 |
| BMI (kg/m^2^) | 20.76 [19.23, 22.58] | 20.96 [19.47, 22.86] | 21.30 [20.00, 24.03] | <0.001^a,b,c^ |
| Duration of infertility (years) | 3.00 [2.00, 5.00] | 3.00 [2.00, 5.00] | 3.00 [2.00, 5.00] | 0.376 |
| Infertility type, n (%) |  |  |  |  |
| Primary infertility | 815 (46.0) | 363 (47.2) | 145 (53.3) | 0.08 |
| Secondary infertility | 956 (54.0) | 406 (52.8) | 127 (46.7) |  |
| TSH (µIU/mL) | 1.62 [1.23, 2.02] | 3.07 [2.76, 3.51] | 5.06 [4.54, 6.14] | <0.001^a,b,c^ |
| FT4 (ng/dL) | 1.25 [1.15, 1.36] | 1.24 [1.13, 1.34] | 1.21 [1.12, 1.31] | <0.001^c^ |
| FT3 (pg/mL) | 2.93 [2.71, 3.14] | 2.98 [2.76, 3.23] | 3.00 [2.76, 3.21] | <0.001^a,c^ |
| TPOAb (IU/mL) | 5.95 [1.51, 11.72] | 7.31 [2.41, 13.89] | 9.18 [2.92, 40.10] | <0.001^a,b,c^ |
| TgAb (IU/mL) | 12.16 [4.50, 22.18] | 13.41 [6.80, 34.15] | 14.34 [6.62, 137.15] | <0.001^a,c^ |
| Positive TPOAb, n(%) | 164 (9.3) | 97 (12.6) | 72 (26.5) | <0.001^a,b,c^ |
| Positive TgAb, n(%) | 196 (11.1) | 131 (17.0) | 73 (26.8) | <0.001^a,b,c^ |
| Positive TAI, n(%) | 246 (13.9) | 150 (19.5) | 86 (31.6) | <0.001^a,b,c^ |
| LH (IU/L) | 4.85 [3.69, 6.34] | 5.02 [3.90, 6.57] | 4.77 [3.59, 6.45] | 0.065 |
| E2 (pg/mL) | 35.00 [26.00, 47.30] | 34.00 [26.20, 48.00] | 32.60 [24.37, 45.82] | 0.127 |
| AMH (ng/mL) | 3.44 [1.96, 5.58] | 3.33 [1.91, 5.23] | 2.73 [1.41, 4.67] | <0.001^b,c^ |
| AFC (n) | 10.00 [6.00, 14.00] | 10.00 [6.00, 14.00] | 9.00 [6.00, 14.00] | 0.697 |
| FSH (mIU/mL) | 6.72 [5.82, 7.98] | 6.93 [5.90, 8.14] | 7.04 [6.08, 8.12] | 0.057 |
| Prevalence of AMH<1.2ng/mL, n(%) | 204 (11.5) | 104 (13.5) | 49 (18.0) | 0.008^c^ |
| Prevalence of AFC<5, n(%) | 214 (12.1) | 115 (15.0) | 42 (15.4) | 0.075 |
| Prevalence of DOR, n(%) | 106 (6.0) | 60 (7.8) | 28 (10.3) | 0.017^c^ |

All data is presented as median [25th percentile, 75th percentile] or n (%).

Superscripts indicate significant differences between ^a^ TSH<2.5µIU/mL and 2.5µIU/mL≤TSH≤4.2µIU/mL, ^b^ 2.5µIU/mL≤TSH≤4.2µIU/mL and SCH, and ^c^ TSH<2.5µIU/mL and SCH.

Kruskal-Wallis rank-sum test is applied for comparisons among three groups of continuous variables, with pairwise comparisons via Dunn’s with Bonferroni correction. Categorical variables are compared using chi-squared test, with pairwise comparisons among multiple groups are conducted using the chi-square partitioning method.

Abbreviations: SCH, Subclinical hypothyroidism; BMI, body–mass index; TSH, thyroid stimulating hormone; FT4, free thyroxine; FT3, free triiodothyronine; TPOAb, thyroid peroxidase antibody; TgAb, thyroglobulin antibody; TAI, thyroid autoimmunity; LH, luteinizing hormone; E2, estradiol; AMH, anti-müllerian hormone; AFC, antral follicle count; FSH, follicle stimulating hormone; DOR, diminished ovarian reserve.
