# Supplementary material for: Subclinical/overt hypothyroidism may be associated with diminished ovarian reserve in infertile women independent of thyroid autoimmunity
Source: Front Endocrinol (Lausanne). 2024 Dec 10;15:1477665. doi: 10.3389/fendo.2024.1477665 (PMC11666349; doi:10.3389/fendo.2024.1477665)
Supplement: Supplementary file 2 [file Table2.docx]

**Supplemental Table 2** Effect of thyroid stimulating hormone levels on DOR.

| **Variables** | **Model 1 OR(95% CI)** | **Model 2 OR(95% CI)** | **Model 3 OR(95% CI)** | **Model 4 OR(95% CI)** |
| --- | --- | --- | --- | --- |
| **TSH levels** |  |  |  |  |
| TSH**<**2.5µIU/mL | Ref |  | Ref | Ref |
| 2.5µIU/mL≤TSH≤4.2µIU/mL | 1.329(0.957,1.846) |  | 1.330(0.957,1.849) | 1.308(0.935,1.829) |
| SCH | 1.803(1.164,2.792)^*^ |  | 1.806(1.160,2.812)^*^ | 1.887(1.193,2.983)^*^ |
| **Thyroid autoimmunity** |  |  |  |  |
| TAI(-) |  | Ref | Ref | Ref |
| TAI(+) |  | 1.069(0.731,1.563) | 0.989(0.673,1.454) | 0.924(0.624,1.370) |
| **Basic clinical characteristics** |  |  |  |  |
| Female age (years) |  |  |  | 1.220(1.164,1.277)^*^ |
| BMI (kg/m^2^) |  |  |  | 1.026(0.975,1.081) |
| Duration of infertility (years) |  |  |  | 1.017(0.970,1.067) |
| Infertility type |  |  |  |  |
| Primary infertility |  |  |  | Ref |
| Secondary infertility |  |  |  | 0.640(0.468,0.876)^*^ |

**Note**: Model 1: Univariate logistic regression analysis of TSH levels. Model 2: Univariate logistic regression analysis of TAI. Model 3: Adjusted for TAI. Model 4: Model 3, with additional adjustments for female age, BMI, infertility duration, infertility type.

**P* value<0.05

Abbreviations: DOR, diminished ovarian reserve; SCH, Subclinical hypothyroidism; TAI, thyroid autoimmunity.
